# Pericallosal artery aneurysm – Case report, literature review and management outcome

**DOI:** 10.1016/j.ijscr.2020.02.022

**Published:** 2020-02-13

**Authors:** O.A. Badejo, A.A. Adeolu

**Affiliations:** a Department of Neurological Surgery, University College Hospital, Ibadan, Nigeria; b Division of Neurological Surgery, Department of Surgery, College of Medicine, University of Ibadan, Nigeria

**Keywords:** Ruptured aneurysm, Pericallosal artery, Clipping, Case report

## Abstract

**Introduction:**

Pericallosal artery aneurysms are rare. Very few cases of this vascular anomaly have been published from West Africa. We report the first case of a ruptured pericallosal artery (PCA) aneurysm managed in a Nigerian neurosurgical facility, with the aim to add to the limited documentation on vascular brain lesions in our sub-region. The management outcome of the index patient and a literature review on these unusual aneurysms were also discussed.

**Case report:**

A middle-aged known hypertensive woman who presented with clinical features of a WFNS grade I subarachnoid hemorrhage (SAH). A plain cranial computerized tomography (CT) scan revealed SAH, a supracallosal intracerebral hematoma and intraventricular hemorrhage. Cranial computerized tomography angiography (CTA) showed a small right pericallosal artery aneurysm, which was treated (with clipping via an interhemispheric approach) in a resource-constrained neurosurgical facility. The patient has remained well over a six-year follow-up period.

**Discussion:**

PCA aneurysms have a high tendency to bleed compared with other supratentorial intracranial aneurysms in spite of their small size. Microsurgical approach, although difficult, is an effective treatment option for these rare aneurysms.

**Conclusion:**

Surgical clipping remains a safe and useful treatment option for pericallosal artery aneurysms in a low-resource neurosurgical facility.

## Highlights

• Pericallosal artery aneurysms are rare.
• They have a higher rupture rate than other anterior circulation intracranial aneurysms.
• These vascular lesions often present with pericallosal intracerebral hematoma.
• Surgical approaches to these aneurysms are associated with a high complication rate.
• Successful management is possible in our low-resource neurosurgical facility.

## 1 Introduction

Pericallosal artery aneurysms (PCAs) are rare, constituting about 2–9% of all intracranial aneurysms (IAs) and about 4% of ruptured ones [1,2]. There is no previously documented case report of this vascular anomaly in Nigerian literature. They are typically saccular, wide-necked and despite their small sizes, are more prone to rupture than other types of anterior circulation IAs [3,4]. The incidence rate of PCAs is higher in individuals with unpaired or azygous pericallosal arteries compared to the general population [5]. Rupture of pericallosal artery aneurysms results in intracerebral hematoma (in about 50% of cases), usually located in the frontal lobe (unilateral or bilateral), anterior interhemispheric fissure, pericallosal cistern, close to the corpus callosum (genu or along its dorsal surface) or cingulate gyrus [6]. Surgical approaches to these rare aneurysms are associated with higher rates of morbidity and mortality compared to other supratentorial aneurysms [4,7]. We describe the operative management of a pericallosal artery aneurysm in a middle-aged female patient treated in a resource-constrained neurosurgical facility. This article has been written in line with the SCARE criteria [8].

### 1.1 Patient information

A fifty-five-year old right-handed Nigerian female petty-trader who presented with sudden severe headache of two days duration (on referral by the neurology team). There was an associated history of vomiting, transient loss of consciousness, neck pain, nuchal stiffness and bisphincteric incontinence. There was no preceding history of trauma. She had a similar occurrence four weeks prior to presentation. She had been diagnosed with systemic hypertension five years earlier but had poor compliance with her antihypertensive medications. There was no past history of surgery in this patient, no family history of a similar illness and no social history of cigarette smoking, alcohol consumption or substance abuse.

### 1.2 Clinical findings

She had normal general examination findings. Her blood pressure at the initial review was 190/100 mmHg. Other vital signs were normal in this patient. She was fully conscious, had positive signs of meningeal irritation and no cranial nerve or sensorimotor deficit. No abnormality was detected in the rest of the neurologic and systemic examination.

### 1.3 Timeline

There was a delay of four weeks from the initial presentation to definitive diagnosis and another four weeks from diagnosis to surgical intervention due to financial constraints.

### 1.4 Diagnostic assessment

**Fig. 1:**
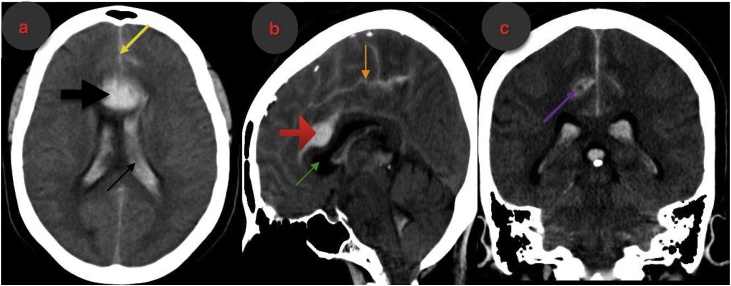
(a–c): Non-contrast Cranial Computerized Tomography Scan. 1a- Axial cut; note the interhemispheric SAH (yellow arrow), ICH (thick black arrow) and intraventicular hematoma (thin black arrow). 1b- Sagittal reconstruction; note the proximity of the ICH (red arrow) to the body of the corpus callosum (green arrow) and its compression and downward displacement by this clot. The orange arrow shows the SAH. 1c- Coronal reconstruction showing SAH and intraventricular hematoma (IVH). Note that the parasagittal SAH is thicker on the right compared to the left (purple arrow).

**Fig. 2:**
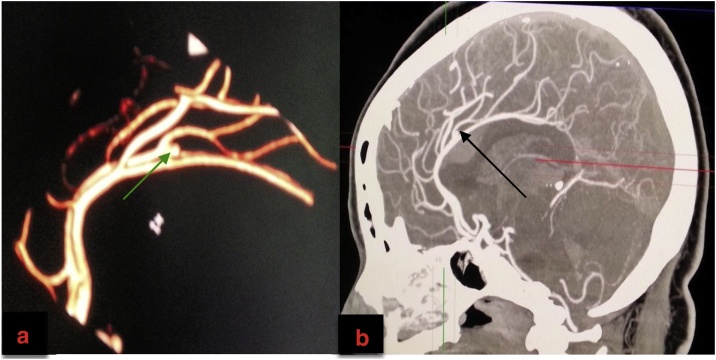
a and b: Cranial Computerized Tomography Angiography. 2a: 3-D Reconstruction (green arrow on the right pericallosal saccular aneurysm). 2b: Sagittal Reconstruction (black arrow showing the same aneurysm).

We made a clinical diagnosis of aneurysmal subarachnoid haemorrhage (WFNS grade I). Other differential diagnosis considered in this patient included a ruptured arteriovenous malformation, an intracranial tumour with intralesional bleed and a haemorrhagic cerebrovascular accident. Cranial computerized tomography (CT) scan revealed ICH superior to the body of the corpus callosum, intraventricular extension of this bleed and interhemispheric/parasagittal subarachnoid haemorrhage (Fisher grade IV SAH) (Fig. 1a–c). Cranial CT angiography outlined a small right pericallosal artery aneurysm (Fig. 2a and b).

### 1.5 Therapeutic intervention

Her pre-intervention treatment considerations encompassed gradual blood pressure control, administration of Nimodipine (a calcium-channel blocker) to prevent vasospasm, administration of stool softener/anti-emetic agents (to reduce the risk of rebleed), and use of non-phamarcologic venous thromboembolism prevention techniques.

**Fig. 3:**
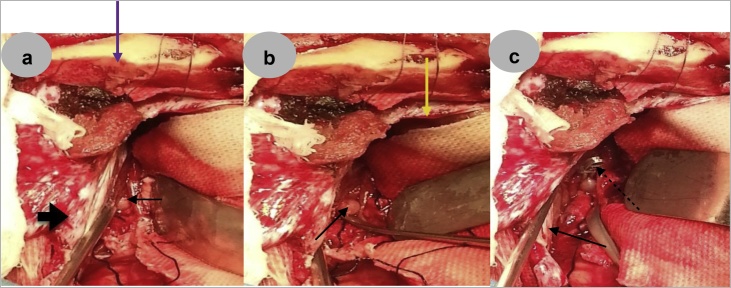
a–c: Intraoperative images. 3a: Bony edge (purple arrow), dural flap based on the superior sagittal sinus (thick black arrow), dome of the aneurysm (thin black arrow), retractor parting the brain away from the midline after an interhemispheric dissection. 3b: Dural edge (yellow arrow), aneurysm sac (black arrow). 3c: Falx cerebri (thin black arrow), aneurysm clip (broken black arrow).

She underwent a right frontoparietal parasagittal craniotomy, interhemispheric dissection and clipping of the aneurysm (Fig. 3a–c) as endovascular coiling was not available in our country as a treatment option. The procedure was performed by an experienced consultant neurosurgeon who was assisted by two trainees. It was done the under general anesthesia in the supine position (with the head elevated 30° in the neutral position) and was well tolerated by the patient. She was nursed in the intensive care unit for the first 24 h post-surgery and was subsequently transferred to the ward. She had an uneventful post-operative course and was discharged home two weeks post-operatively.

### 1.6 Follow-up and outcome

The patient was followed-up for six years. She has maintained functional independence, returned back to her pre-morbid vocation and has not had any episode of rebleed or new neurologic deficit. Post-operative angiographic study (although requested) has not been done on account of financial challenges.

## 2 Discussion

Intracranial aneurysms (IAs) are considered to be uncommon in Africans. Various authors have challenged this viewpoint over the last two decades [9]. However, there is a paucity of reports of this pathology in West African literature, particularly in Nigeria, where vascular brain lesions are underreported. Our centre has experienced an upsurge in the diagnosis of IAs in the last decade owing largely to the increased availability of neuro-diagnostic facilities in our sub-region. We report a rare aneurysm managed in our hospital, with the aim to add to the limited data on IAs in West Africa. This represents the first case report of a pericallosal aneurysm in a Nigerian.

Pericallosal aneurysms are ACA aneurysms arising distal to the origin of the anterior communicating artery (A2-A5) [3,10]. Although these are usually small berry aneurysms, giant and fusiform cases have been reported in the literature [4,[11], [12], [13]]. The typical location for these aneurysms is at the bifurcation of the pericallosal and callosomarginal arteries but on rare occasions, they could be found proximal or distal to this site [4]. Congenital aetiology is the most commonly implicated but PCAs at the aforementioned unusual locations have been linked to vascular anomalies, trauma and infection as possible causes [11]. Iatrogenic cases of PCAs exist [13]. Despite their small sizes, they are fraught with a high tendency for bleeding and as such, higher morbidity and mortality rates compared to other types of ICA aneurysms if left untreated [4,7]. The multiplicity of PCAs is not uncommon and has been reported to be >40% in some studies [4,11]. It commonly has a female preponderance, but a higher frequency in the male gender has been published in the literature [1,2,4]. The age range varies in different series (from the second to the eighth decade of life), but these aneurysms can be found in younger and older individuals [1,4,7,10,13]. Our patient’s demography (sex, age) and aneurysm characteristics (size, site, morphology, the high tendency for rebleeding) fit into those described for PCAs.

Clinical presentation is usually that of a spontaneous intracranial bleed. However, PCA related thromboembolic events, and incidental findings have been documented by previous authors [2]. Ruptured pericallosal artery aneurysms could present as ICH, SAH or bleeding around the corpus callosum all of which were present in the index patient. Co-existence with an arteriovenous malformation as well as an acute subdural hematoma resulting from a ruptured pericallosal artery aneurysm have been reported in the literature [14,15]. Other unusual findings in previous reports include mirror or kissing PCA aneurysms, tracking of the bleed to the contralateral side via the corpus callosal commissural tracks, mimicry of a brain tumour, intratumoural bleed associated with intracranial meningioma (intralesional PCA) and concurrence with a glioblastoma [3,4,10,[16], [17], [18], [19]]. Association of these aneurysms with corpus callosal lipoma has been reported as well [12]. Asides clinical features of SAH, symptoms/signs include paraparesis, lower limb monoparesis, hemiparesis, lower limb sensory abnormalities, sphincteric dysfunction, behavioural abnormalities and rarely, seizures [1,11].

The proximity of a non-traumatic ICH to the corpus callosum on plain cranial CT scan should raise the suspicion of a ruptured PCA aneurysm origin. Our patient's neuroimaging findings were classical of a ruptured pericallosal artery aneurysm. Diagnosis can be confirmed with conventional angiography (cranial CT angiography, MR angiography, or digital subtraction angiography). Further evaluation with a four-vessel angiography should be done to rule out other synchronous aneurysms [11]. These neuroimaging modalities are useful for establishing the diagnosis, defining the anatomy of the parent vessels, surgical planning and detecting other associated anomalies or lesions.

The primary goals of treatment are early identification and intervention to prevent re-rupture and facilitate the aggressive management of vasospasm [6,7,11]. The neurosurgical procedure can be microsurgical or endovascular. Conservative care is not advisable because of the high tendency for repetitive bleeding in these types of aneurysms [4,11]. Of note is the fact that our patient bled twice within a four-week interval before her definitive surgical intervention. The choice of surgical approach depends on the presence or absence of multiple aneurysms and the location of the aneurysm along the course of the pericallosal artery [7]. Microsurgical approaches to PCAs are daunting because of the restricted surgical corridor, possible need to sacrifice a bridging vein to enhance access, interhemispheric adhesions, difficulty in achieving control of the parent artery, lack of surgical landmarks, orientation of the dome of the aneurysm toward the surgeon and the high risk of intraoperative rupture [4,7,11]. Possible approaches for the treatment of PCAs include pterional, anterior intehemispheric (as in our patient), frontobasal interhemispheric and sub-frontal [1,7,11,20]. Intervention can be staged in multiple aneurysms [4]. Deterrents to endovascular therapy include the small size, broad base of these aneurysms, distal location and the potential for compromise of the parent vessel with its attendant clinically debilitating consequences [4]. However, while a good outcome of endovascular therapy has been reported in many centres especially in recent series, some authors believe that microsurgical treatment is superior in terms of safety, immediate benefits and long-term efficacy [4,10].

Prognostic factors of PCAs includes severity of the SAH grade at the initial review, the presence of ICH, recurrent bleeding before operative care, timeliness of the surgical intervention, the presence of hydrocephalus pre-operatively, the patient’s age, multiplicity, and the surgeon’s experience [3,4,11]. Our patient had a successful clipping, had no surgery-related complication and has not rebled over a follow-up period of six years.

## 3 Conclusion

Surgical clipping remains a safe and useful treatment option for pericallosal artery aneurysms in a low-resource neurosurgical facility.

## Sources of funding

None.

## Ethical approval

Ethical approval is not required for case reports at my institutions (College of Medicine, University of Ibadan and University College Hospital, Ibadan, Nigeria).

## Consent

A written informed consent was obtained from the patient for the publication of this case report and the accompanying images. A copy of this written consent is available for review by the Editor-in-Chief of this journal on request.

## Author contribution

Prof. Augustine A. Adeolu conceptualized this case report, provided the images used, supervised every aspect of this work and edited/approved the final version.

Dr Oluwakemi A. Badejo drafted the manuscript.

## Registration of research studies

Not applicable.

## Guarantor

Dr Oluwakemi A. Badejo and Prof. Augustine A. Adeolu.

## Provenance and peer review

Not commissioned, externally peer-reviewed.

## Patient’s perspective

The patient is grateful for a successful treatment and happy to reunite with her family and to be able return back to her previous daily routine.

## Declaration of Competing Interest

None.
